# Outpatient Pulmonary Rehabilitation in Patients with Persisting Symptoms after Pulmonary Embolism

**DOI:** 10.3390/jcm9061811

**Published:** 2020-06-10

**Authors:** Stephan Nopp, Frederikus A. Klok, Florian Moik, Milos Petrovic, Irmgard Derka, Cihan Ay, Ralf Harun Zwick

**Affiliations:** 1 Clinical Division of Haematology and Haemostaseology, Department of Medicine I, Medical University of Vienna, 1090 Vienna, Austria; stephan.nopp@meduniwien.ac.at (S.N.); Florian.moik@meduniwien.ac.at (F.M.); 2 Department of Thrombosis and Hemostasis, Leiden University Medical Center, 2333 ZA Leiden, The Netherlands; f.a.klok@LUMC.nl; 3 Outpatient Pulmonary Rehabilitation, Therme Wien Med, 1100 Vienna, Austria; petrovic@gmx.at (M.P.); Irmgard.Derka@thermewien.at (I.D.); ralfharun@hotmail.com (R.H.Z.); 4 I M Sechenov First Moscow State Medical University, 119146 Moscow, Russia

**Keywords:** pulmonary rehabilitation, pulmonary embolism, outpatient, dyspnea, exercise training

## Abstract

**Background:** Patients with pulmonary embolism (PE) may suffer from long-term consequences, including decreased functional capacity. Data on pulmonary rehabilitation (PR) in patients with PE are scarce, and no data on outpatient PR are available so far. **Methods:** We analyzed data of 22 PE patients who attended outpatient PR due to exertional dyspnea. Patients underwent a multi-professional 6-week PR program. The primary outcome was change in 6-min walk test (6MWT). Secondary outcomes included changes in strength and endurance tests. To assess long-term benefits, follow-up was performed a median of 39 months after PR. **Results:** Patients started PR a median of 19 weeks after the acute PE event. Their median age was 47.5 years, 33% were women and all presented with NYHA (New York Heart Association) class II and higher. After PR, patients showed significant and clinically relevant improvements in 6MWT (mean difference: 49.4 m [95% CI 32.0−66.8]). Similarly, patients increased performance in maximum strength, endurance and inspiratory muscle strength. At long-term follow-up, 78% of patients reported improved health. **Conclusion:** We observed significant improvements in exercise capacity in PE patients undergoing outpatient PR. The majority of patients also reported a long-term improvement in health status. Prospective studies are needed to identify patients who would benefit most from structured PR.

## 1. Introduction

Pulmonary embolism (PE) is an acute manifestation of venous thromboembolism (VTE), which is potentially life-threatening. The incidence of PE has increased over time, while its case fatality rate has declined [1,2]. A significant proportion of survivors of acute PE are at risk of disabling long-term sequela, commonly referred to as post-PE syndrome [3,4]. Post-PE syndrome, with its most severe manifestation being chronic thromboembolic pulmonary hypertension (CTEPH), is characterized by persistent dyspnea, chronic (cardiopulmonary) functional limitations and decreased quality of life. The incidence of post-PE syndrome has been reported to reach up to 50% and up to 4% of PE patients may manifest with CTEPH [5,6,7].

While many studies in PE survivors have focused on investigating the risk of recurrence, presence of residual thrombosis and right ventricular strain [8], few studies have examined persistent dyspnea and functional outcomes. Of note, reduced physical performance, assessed with the 6-min walk test (6MWT), has been reported in approximately half of all patients with PE, which again is associated with decreased health-related quality of life [9,10,11]. Similarly, patient-reported outcome measures have indicated an acute decline in physical function after PE and a study on massive or submassive PE suggests that overall deconditioning rather than cardiopulmonary insufficiency imposes persistent symptoms [12,13]. Taken together, this highlights the need for measures to improve functional deficits in patients with PE.

After acute myocardial infarction, participation in a cardiopulmonary rehabilitation program is recommended to increase functional capacity and quality of life, and decrease cardiovascular mortality [14]. However, in patients with PE, evidence for rehabilitation is scarce and participation in rehabilitation programs after PE is not routine. One retrospective analysis and one prospective study proved feasibility and safety of inpatient pulmonary rehabilitation (PR) in PE survivors [15,16], but no study has evaluated the effect of outpatient PR in patients with PE so far. Only recently, a guideline for exercise training and rehabilitation was released for patients with chronic pulmonary hypertension, including CTEPH [17]. Whether a broader population of patients with PE and persisting symptoms or functional limitations would benefit from rehabilitation is yet unknown.

The aim of this study was to evaluate the effect of structured outpatient PR in a population of PE patients with persisting symptoms. Our objectives were to investigate changes in exercise capacity (e.g., 6MWT) after completion of PR and to evaluate long-term effects on quality of life, functional status and overall health status.

## 2. Methods

### 2.1. Study Design and Patient Cohort

We conducted a retrospective single-center cohort study of all consecutive patients with PE who were referred for and completed PR at the Outpatient Pulmonary Rehabilitation Unit of the Therme Wien Med (Vienna, Austria) between 1 January 2012 and 30 September 2019. Only patients with a history of PE and exertional dyspnea as reason for admission to PR were eligible for this study. Overall, 24 eligible patients were identified. Two of them had to be excluded due to early termination of the rehabilitation program: one patient was admitted to hospital due to a suspected tumor and one was pregnant and underwent abortion. Therefore, the final cohort included in this study consisted of 22 patients with PE.

The study consisted of two parts. In the first part, we evaluated the outcomes and complications of the PR in terms of improvements regarding exercise capacity at completion of the rehabilitation program compared to baseline assessment at the beginning of PR. In the second part of the study, we contacted every patient for a follow-up to perform long-term evaluation of PR with regard to quality of life, functional status and overall health status.

All patients included in the analysis provided written informed consent for analysis of their rehabilitation data. The study was conducted according to the principles of the Declaration of Helsinki.

### 2.2. Rehabilitation Procedures

At admission to outpatient PR, a detailed medical history was taken and information on demographics, comorbidities, medications, history of smoking and PE-relevant data (triggering events, method of diagnosis, site and location of PE, type of treatment, etc.) were recorded in all patients.

Patients underwent a multi-professional and individualized rehabilitation according to the Austrian guidelines for outpatient PR [18]. Three times a week for 3 to 4 h, participants completed endurance, strength and inspiratory muscle training over at least 6 weeks. Additionally, the program consisted of individualized patient education, which included disease- and medical treatment-specific seminars, nutritional counseling, and smoking cessation sessions. A detailed description of the program is available in the Supplementary Materials (Supplementary S1).

### 2.3. Outcome Variables

The primary outcome of the present analysis was change in 6-min walk distance (6MWD) after completion of PR program, as the 6MWT (6-min walk test) is well established for testing physical performance ability in patients with cardiopulmonary disease [19]. Assessment of 6MWT was performed on a 30 m walking course according to the standardized protocol of the American Thoracic Society [20]. To determine meaningful change in 6MWD we used a minimal clinical important difference (MCID) of 30.5 m, which was based on a systematic review on 6MWT in adults with cardiopulmonary diseases [21].

Secondary outcome variables were changes in physical assessments including maximal workload (Wmax) on cycle ergometer in watt, constant work rate test at 70% of Wmax (CWR70%) in minutes, 1-min sit-to-stand test (1-MSTST), upper and lower extremity strength in kilogram (kg) and maximal inspiratory muscle strength (Pimax) in mbar. Moreover, dyspnea during cycle ergometer was assessed via modified Borg-scale (1 = no exertion, 10 = maximal exertion) [22]. Additionally, changes in forced expiratory volume in one second (FEV1) and vital signs (heart rate, blood pressure) at completion of the PR program were recorded. Complications including bleeding events, VTE recurrence, myocardial infarction and unexpected hospital admissions were recorded.

### 2.4. Long-Term Follow-Up

In November 2019, every patient was contacted via phone. A structured follow-up interview was performed, which included assessment of functional limitations with the post-VTE functional status scale [23], patient-reported health status (patients were asked to compare their current health status with that before PR ranging from “much better” to “much worse”), VTE recurrence, comorbidities, anticoagulation regimen, hospital admissions, smoking habits and body mass index (BMI). Additionally, patients were asked to report their quality of life measured with the Short Form-36 Health Survey (SF-36), which was sent to study participants by mail [24]. The SF-36 is a well-known health-related quality of life questionnaire, divided into 8 subscales with higher values representing better quality of life. Subscales of the SF-36 were calculated and compared to a reference population norm derived from the literature [25].

### 2.5. Statistical Analysis

Patient demographics, clinical characteristics, and follow-up data are summarized as median and 25th–75th percentile (i.e., interquartile range [IQR]), or frequencies and proportions, as appropriate. 

The primary outcome variable (6MWD) was assessed by comparing pre- and post-rehabilitation measurements by means of a paired samples *t*-test. Normality of the distribution of differences in measurements were assessed graphically by Q-Q-plots and histograms. Clinically meaningful changes of the 6MWD were defined as a difference of 30.5 m [21]. In addition, individual 6MWD was compared to a predicted walking distance, calculated with a standard reference equation (variables: height, weight, age) [26].

Secondary outcome parameters have been assessed by comparing pre- and post-rehabilitation measurements by means of paired samples *t*-test or paired samples rank-sum test, depending on distribution of mean-differences. Again, normality was assessed graphically by Q-Q-plots and histograms. Primary and secondary outcome parameters are presented as means and standard deviation (±SD).

Quality of life assessments are presented utilizing the prespecified specific subscales within the SF−36 questionnaire and were compared to the reference population norm by using unpaired samples *t*-test [25].

Statistical analysis was performed using SPSS statistical software (IBM Corp. Released 2017. IBM SPSS Statistics for Windows, Version 25.0. IBM Corp.: Armonk, NY, USA).

## 3. Results

### 3.1. Study Population

Twenty-two patients with PE (7 (32%) women and 15 (68%) men) with a median age of 47.5 (IQR: 42.5−54.3) years and a median BMI of 32.4 (27.8–37.4) kg/m² were included in this study. PE was diagnosed in all patients with computed tomography pulmonary angiography and treated in 10 (50%) cases with phenprocoumon, 5 (25%) cases with rivaroxaban, 4 (20%) cases with apixaban, and one (5%) case with edoxaban. In two patients no information on the treatment regimen could be obtained. PE was the first VTE event in 15 (68.2%) cases. In 7 (31.8%) cases it was a recurrent VTE event and of those, three had already experienced a PE before. Patients started the PR program a median of 19 (14–37) weeks after diagnosis of PE and median duration of PR was 6 (6–10.5) weeks. At admission, all patients reported dyspnea classified as New York Heart Association (NYHA) class II or higher. Baseline characteristics at admission to PR and detailed information on PE are summarized in Table 1. No malignancies were recorded in this cohort. Three patients presented with right ventricular strain in the initial echocardiogram at PE diagnosis, while only one patient showed right ventricular strain in the obligatory echocardiogram prior to rehabilitation, although no other signs of CTEPH were seen. In all patients, normal left ventricular function was described. Hence, CTEPH was considered excluded in all patients. No patient relied on oxygen therapy at baseline or during rehabilitation. None of the patients desaturated below 90%, no usage of oxygen and no serious adverse events (bleeding events, VTE recurrence, myocardial infarction, unexpected hospital admissions) were recorded during the entire duration of PR.

### 3.2. Changes in Outcome Variables

**Figure 1:**
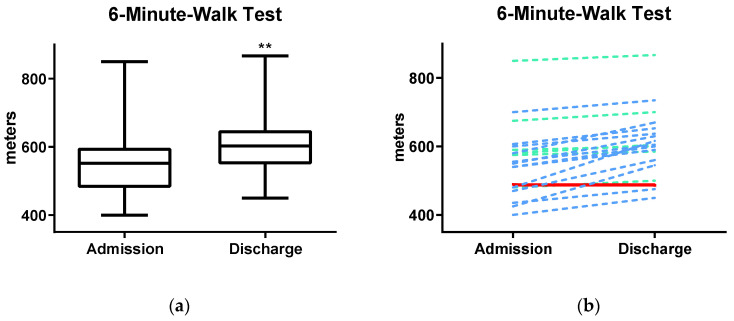
(**a**) Improvements in 6MWT (6-min walk test) at completion of pulmonary rehabilitation. 6MWT improved significantly after completion of pulmonary rehabilitation. (**b**) Individual patients’ results for 6MWT at admission and discharge are presented on the right. Thirteen patients improved above a minimal clinical important difference of 30.5 m (blue line). Six patients improved but were below the minimal clinical important difference (green line). One patient did not improve (red line); Abb.: 6MWT, 6-min walk test; ** = *p* < 0.001, paired samples *t*-test.

The results of the primary outcome (6MWD) are presented in Figure 1a and Table 2. Mean 6MWD at baseline was 556 (±105) m, which accounts for 94% of the mean predicted value (calculated by height, weight, age) [25]. After PR, patients presented with significant and clinically relevant changes of the 6MWD, which increased in mean to 605 (±96) m (mean difference (MD): 49.4 m [95% confidence interval (CI) 32.0, 66.8]): This corresponds to 102% of their mean predicted walking distance. While all but one improved in their performance, 65% of the tested patients also reached a minimal clinical important difference of 30.5 m (Figure 1b).

**Figure 2:**
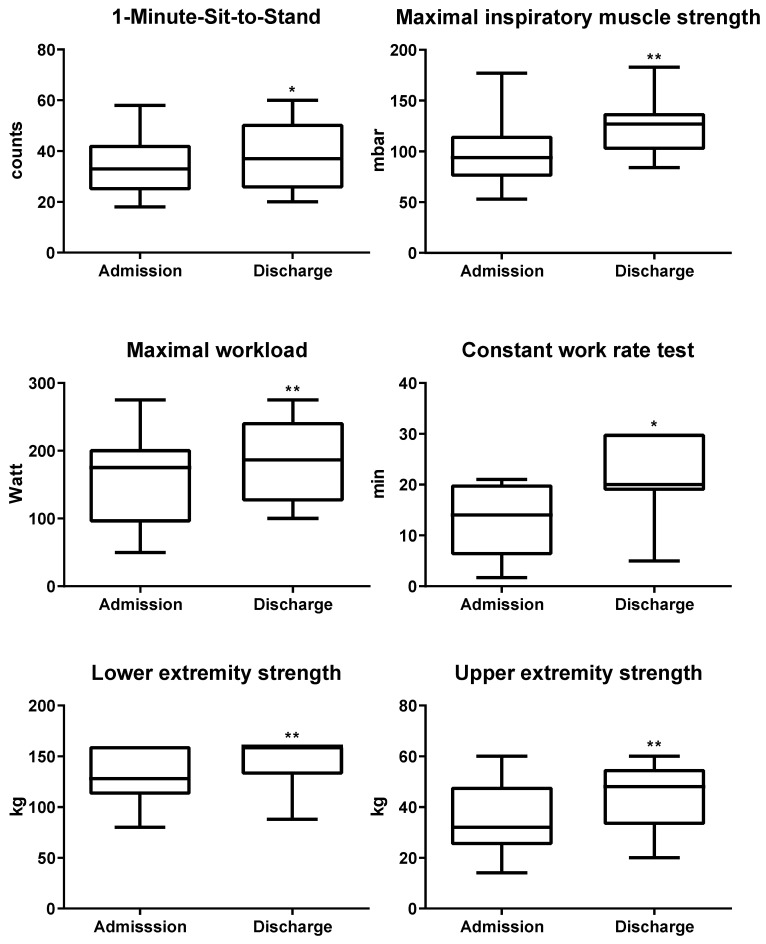
Improvements in secondary outcome parameters after completion of pulmonary rehabilitation. Patients improved significantly in 1-MSTS, maximal inspiratory muscle strength, maximal workload, constant work rate, lower and upper extremity strength; * = *p* < 0.05; ** = *p* < 0.001, paired samples *t*-test.

All six secondary exercise outcome parameters showed similar improvements after completion of PR (Table 2 and Figure 2). For analysis of CWR70% and lower extremity strength, 4 and 5 patients, respectively, had to be excluded from analysis. They already reached the maximum measurable outcome at admission, without the possibility of further measurable improvement during PR. For the record, they all reached the maximum outcome at discharge again. At discharge, 1-MSTST performance increased from 35 (±12.6) to 39 (±14.2) times (MD: 3.9 [95%CI 0.4, 7.5]). All patients raised their inspiratory muscle strength measured by Pimax (94.7 (±30.4) to 125.2 (±27.0) mbar; MD: 30.5 [95%CI 23.2, 37.8]). Both cycle ergometer tests, peak performance (156.8 (±63.2) to 188.5 (±57.1) watt; MD: 31.7 [95% CI 19.9, 43.5]) and endurance capacity (CWR70%, 12.7 (±6.7) to 21.2 (±7.7) min; MD: 8.5 [95% CI 3.8, 13.2]) improved greatly. In addition, the BORG dyspnea scale slightly decreased during endurance testing (5.3 (±1.7) to 4.2 (±1.8), MD: −1.1 [95% CI −2.5, 0.3]), while it remained unchanged despite higher workload during peak performance tests (6.3 (±2.2) to 6.0 (±2.3), MD: −0.3 [95% CI −1.6, 1.1]). Furthermore, participants significantly gained strength in upper (34.9 (±12.6) to 44.5 (±11.8) kg; MD: 9.6 [95% CI 5.3, 14.0]) and lower extremities (117 (±17.9) to 146.9 (±16.5) kg; MD: 29.9 [95% CI 23.1, 36.8]). 

Weight, vital signs and FEV1 at admission and discharge are presented in Table 3; here, no significant changes were observed.

### 3.3. Long-Term Follow-Up

A follow-up to perform long-term evaluation of PR could be performed in 20 (91%) patients. Patients were contacted a median of 38.9 [14.6–56.5] months after PR. They reported a median BMI of 31.8 [27.9–36.1] kg/m^2^ and a median weight of 101.5 (mean 98.4) kg. During follow-up, one female patient suffered from recurrent PE, which occurred post-partum. She mentioned poor adherence in taking low-molecular-weight heparin, which was prescribed during pregnancy and post-partum period for VTE prophylaxis. Two patients reported admission to hospital: one had myocardial bypass grafting surgery, while the other suffered from a viral respiratory infection. At follow-up, 12 (60%) patients were still on oral anticoagulation to prevent VTE recurrence and 8 (40%) had stopped anticoagulation treatment. No change in smoking status was recorded.

**Figure 3:**
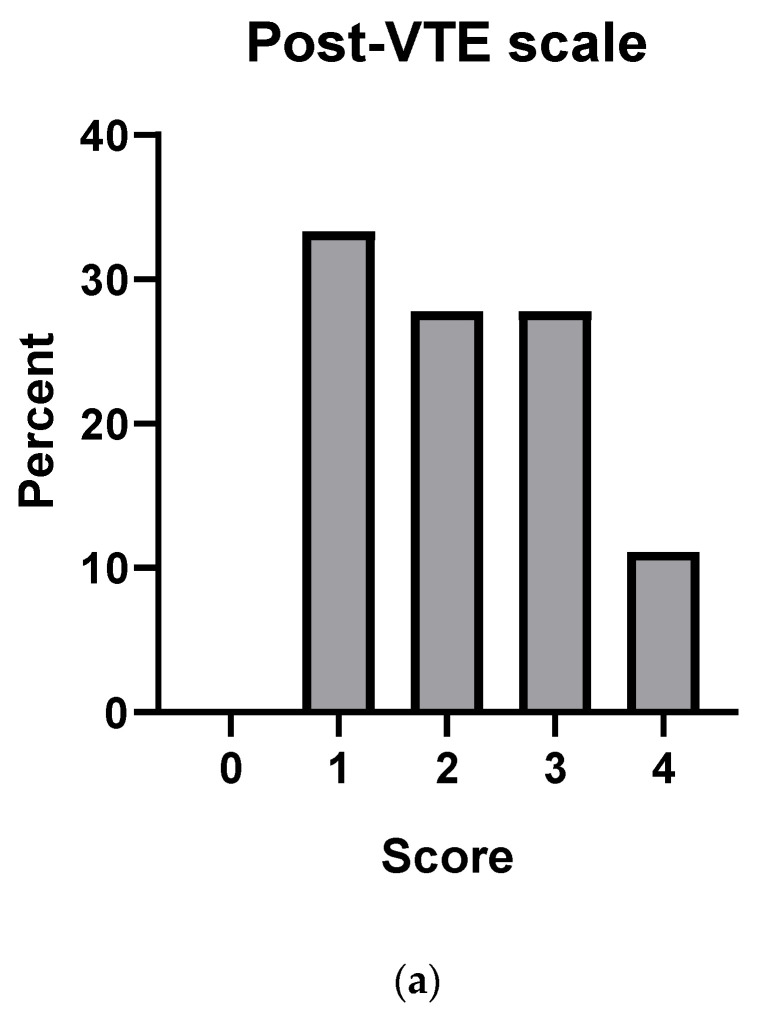

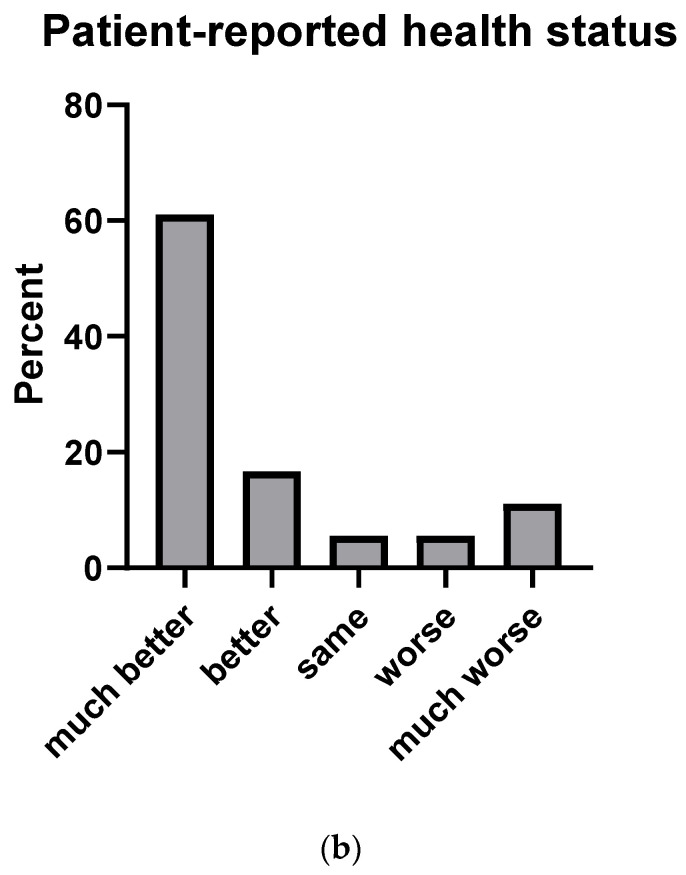
(**a**) Post-VTE (venous thromboembolism) functional status scale and patient-reported health status at long-term follow up. 0, No significant functional limitations; 2, Slight functional limitations; 3, Moderate functional limitations; 4, Severe functional limitations. (**b**) 78% reported much better or better health status compared to before admission to pulmonary rehabilitation.

At long-term follow-up, patient-reported health status and functional limitations were assessed. Eleven (61%) patients reported no significant functional limitations, 7 (39%) patients indicated different degrees of functional limitations measured by post-VTE functional status scale, and two provided no data regarding functional limitations at follow-up (Figure 3a). In 14 (78%) patients the general health status improved after PR, in 3 (17%) patients the health status deteriorated during follow-up and 3 patients did not report information on their general health status (Figure 3b).

Eighteen (82%) patients completed the quality of life assessment (SF-36 questionnaire). All subscales of the SF-36 showed decreased health-related quality of life compared to the age group of the reference population norm reported in the literature [25]. Details are presented in Table 4.

## 4. Discussion

We investigated the effect of outpatient PR in patients suffering from exertional dyspnea after PE. At completion of the outpatient PR program, patients had improved significantly in all exercise tests, such as 6MWT, endurance and peak physical performance, inspiratory muscle strength and upper and lower extremity strength. Exercise training was well tolerated, and no adverse effects or complications occurred during outpatient PR. At follow-up, 78% of all patients reported a long-term benefit in general health status compared to the time before PR. 

Rehabilitation has been shown to be beneficial in patients with several cardiopulmonary diseases, also including heart failure and chronic obstructive pulmonary disease. More recent studies have focused on PR in patients with pulmonary hypertension, especially the subgroup of those with CTEPH [27,28,29]. Two studies evaluated feasibility and safety of PR in VTE patients [15,30] and only one small prospective study investigated outcomes of inpatient PR after PE [16]. However, so far no study has investigated outcomes of outpatient PR in PE patients, which motivated us to perform the present study to expand the available data on PE rehabilitation programs. To measure efficacy of PR programs, 6MWT, dyspnea and quality of life have been identified as the three major outcome parameters [31]. The 6MWT is a simple, frequently used method in cardiopulmonary studies, and a difference of 30.5 m between measurements is considered a minimal clinical important difference [21]. Within 6 weeks of a structured PR program, PE patients of our study significantly improved their mean 6MWD by 49 m. In a meta-analysis of PR in patients with chronic obstructive pulmonary disease, similar improvements (mean 6MWT difference: 48 m) after a 6- to 12-week program were reported [32]. Furthermore, the maximal workload and maximal inspiratory strength of our patients improved from 80% to 96% and 90% to 120% of their age- and sex-adjusted median reference value, respectively [33,34]. Median endurance capacity increased by over 65%, while upper and lower extremity strength improved by 25%. Interestingly, dyspnea, measured with the BORG scale, did not differ significantly. This may be due to the relatively low patient number included in our study or to the fact that different issues such as anxiety or post-traumatic stress disorder were the main determinants of their persisting symptoms. Still, patients reported lower dyspnea levels during endurance testing at discharge.

In PE survivors, quality of life is reduced [35,36]. In accordance with the literature, our findings revealed lower scores in all subscales of the SF-36 compared to a reference population norm, even despite the PR. Compared to other PE cohorts, patients in our study were significantly younger and healthier but had a higher BMI [37,38,39]. Nonetheless, our participants reported similar or worse scores of quality of life compared to other PE cohorts, highlighting the disease burden of our patients [35,36]. As a novel element in this study, we included assessment of functional limitations with the post-VTE functional status scale [23]. Although the majority of patients reported an improvement in their general health status, most patients still mentioned at least some degree of functional limitations at follow-up. To better understand this phenomenon, it is important to explore factors associated with functional limitations in future studies. 

Limitations of the study include the low number of patients and missing values of the index presentation and treatment due to the retrospective nature of data collection. The main limitation, however, is that we are not aware of the selection procedures by PE caretakers to decide on referral for PR. Hence, we do not know why these patients and not other patients were referred. Because of the design of the study, we could not adjust for this selection bias. Interestingly, more men than women underwent PR in our study. This might be explained by the results of the ELOPE study, in which men were at 3-fold increased risk of developing exercise limitations after PE and may, therefore, be referred to PR more often [9]. Furthermore, the very high BMI in this cohort may have been a potential confounder, though we were not able to prove it. Possibly, this study selected a subgroup of post-PE patients who dramatically decreased their physical activity due to post-PE dyspnea resulting in weight gain, which is often observed in clinical practice. However, our findings cannot be generalized to unselected PE patients with persisting symptoms. Nevertheless, the outcomes of outpatient PR in our cohort were highly significant, supporting the effectiveness of an outpatient PR program in patients with the post-PE syndrome.

Follow-up strategies and diagnostic workup to identify patients with post-PE symptoms such as dyspnea and/or functional limitations are now recommended in the latest PE guidelines of the European Society of Cardiology (ESC) developed in collaboration with the European Respiratory Society (ERS) [40]. Assessment of functional limitations with the post-VTE functional status scale could be a novel approach to screen for patients with the need for long-term treatment including PR, although it was mainly designed to quantify the impact of PE diagnosis on long-term functional outcomes [23]. 

Implementation of PR in an integrated model of care after PE is not yet established, as in the case of several cardiopulmonary diseases. Further studies are needed to confirm short- and long-term outcomes and beneficial effects of PR on quality of life and general health status before implementation of PR programs as part of the long-term management of PE survivors with persisting symptoms. Such studies also have to establish the optimal time point of starting PR after PE and to identify groups of patients that would benefit most from PR. Outpatient PR along with other programs with easy access and a low threshold for participation might be an option to reach as many PE survivors as possible.

Taken together, our findings outline the potential beneficial effects of PR in patients suffering from chronic functional limitations after PE. We observed significant improvements at completion of outpatient PR in all physical assessments. At follow-up, the majority of patients reported lasting beneficial effects on their general health status after PR. Future research is required to further demonstrate the effect of PR in PE patients presenting with different grades of persistent dyspnea and functional limitations and to evaluate the relative efficacy of PR programs in this often neglected patient population.

## Figures and Tables

**Table 1:** Patient demographics, PE characteristics and time until pulmonary rehabilitation after PE.

Variable	Patients with Data/Total Number of Patients	Median [IQR] or Count (%)
**Demographics**		
Age (years)	22/22	47.5 [42.5–54.3]
Female	22/22	7 (31.8%)
BMI (kg/m²)	22/22	32.4 [27.8–37.4]
Weight (kg)	22/22	102.5 [87.5–114.8]
**PE characteristics ^†^**		
DVT present at index event	21/22	12 (57.1%)
Triggering events/risk factors		
--Unprovoked	22/22	13 (59.1%)
--Oestrogen use	22/22	4 (18.2%)
--Recent surgery (major/minor)	22/22	3 (13.6%)
--Long travel (>4 h, past 30 days)	22/22	1 (4.5%)
--Benign tumor	22/22	1 (4.5%)
Site of PE		
--Unilateral	20/22	2 (10%)
--Bilateral	20/22	18 (90%)
Location of PE		
--Central	16/22	9 (56.3%)
--Lobar	16/22	2 (12.5%)
--Segmental	16/22	5 (31.3%)
**History of VTE**		
Previous DVT	22/22	4 (18.2%)
Previous PE	22/22	3 (13.6%)
**Comorbidities**		
Charlson Comorbidity Index (CCI)	22/22	0.55 (0–0)
Chronic obstructive pulmonary disease	22/22	2 (9%)
History of acute lymphatic leukemia	22/22	1 (4.5%)
Arterial hypertension	22/22	10 (45.5%)
Active smoker	20/22	3 (15%)
History of smoking	20/22	9 (45%)
**Timeline**		
Time between PE diagnosis and start of rehabilitation (weeks) *	22/22	19 [14–37.25]
Duration of rehabilitation (weeks)	22/22	6 [6–10.50]

† Three patients suffered from recurrent PE. Data for the recurrent event is presented here. Abb.: DVT, deep vein thrombosis; PE, pulmonary embolism; VTE, venous thromboembolism. * In case of recurrent PE, the date of the latter event was taken.

**Table 2:** Exercise parameters at admission to and reassessment at completion of pulmonary rehabilitation ^†^.

Assessment (Measurement)	Patients with Data/Total Number of Patients	Admission	Discharge	Difference (Percent)	*p*-Value
6MWT (m)	20/22	556.1 (±104.8)	605.5 (±96.0)	+49.4 (9%)	<0.001
1-MSTST	14/22	35.1 (±12.6)	39.0(±14.3)	+3.9 (11%)	0.034
Pimax (mbar)	22/22	94.7 (±30.4)	125.2 (±27.0)	+30.5 (32%)	<0.001
Wmax (watt)	18/22	156.8 (±63.6)	188.5 (±57.1)	+31.7 (20%)	<0.001
CWR70% (min) *	14/22	12.7 (±6.7)	21.2 (±7.7)	+8.5 (67%)	0.002
LE strength (kg) #	11/22	117 (±17.9)	146.9 (±16.5)	+29.9 (26%)	<0.001
UE strength (kg)	16/22	34.9 (±12.6)	44.5 (±11.7)	+9.6 (28%)	<0.001

^†^ Plus-minus values are means (±SD); * Four patients had to be excluded from analysis because they already reached the maximum outcome at admission. Hence, no improvement was possible; # Five patients had to be excluded from analysis because they already reached the maximum outcome at admission. Hence, no improvement was possible; Abb.: 1-MSTST, 1-min sit-to-stand-test; 6MWT, 6-min walk test; CWR70%, constant work rate test at 70% of maximal intensity; LE, lower extremity; Pimax, maximal inspiratory pressure; UE, upper extremity; Wmax, maximal workload.

**Table 3:** Changes in vital parameters, weight and FEV1 at completion of pulmonary rehabilitation ^†^.

Vital Parameters and FEV1	Patients with Data/Total Number of Patients	Admission	Discharge	Mean Difference [95% CI]
HR at rest	20/22	89.6 (±19.0)	83.8 (±14.4)	−5.8 [−12.5, 0.9]
HR at max	20/22	142.5 (±18.1)	144.9 (±18.4)	2.4 [−4.1, 8.8]
sysBP at rest (mmHg)	17/22	132.7 (±16.0)	130.8 (±11.2)	−1.9 [−10.4, 6.7]
diaBP at rest (mmHg)	17/22	82.7 (±68.8)	82.9 (±48.9)	0.2 [−4.5, 4.9]
sysBP at max (mmHg)	15/22	190.8 (±39.5)	193.2 (±32.7)	2.4 [−14.7, 19.5]
diaBP at max (mmHg)	15/22	85.3 (±18.4)	86.1 (±19.4)	0.8 [−10.9, 12.5]
Weight (kg)	22/22	99.9 (±19.8)	99.4 (±19.8)	−0.5 [−1.6, 0.6]
FEV1 (liter)	20/22	3.03 (±0.6)	3.07 (±0.6)	0.04 [−0.2, 0,3]

^†^ Plus-minus values are means (± SD); Abb.: HR, Heart rate; sysBP, systolic blood pressure; diaBP, diastolic blood pressure; max, maximal workload; FEV1, forced expiratory volume in one second.

**Table 4:** Subscales of Short Form-36 Health Survey at long-term follow-up ^†^.

	PR Cohort (*n* = 18)	Reference Population Norm Age 40–49 [25]
Physical functioning (PF)	76.7 (±17.2) *	89.5
Role physical (RP)	68.1 (±39.1)	85.5
Role emotional (RE)	72.2 (±40.0)	86.8
Vitality (VT)	50.3 (±19.1) *	60.7
Mental health (MH)	69.6 (±14.8)	72.8
Social functioning (SF)	75.0 (±26.8)	85.6
Bodily Pain (BP)	71.9 (±23.1)	75.3
General health (GH)	50.0 (±23.2) *	69.9

^†^ Plus-minus values are means (±SD); Higher values of the SF-36 subscales reflect better quality of life. * *p* < 0.05.

## Supplementary Materials

The following are available online at https://www.mdpi.com/2077-0383/9/6/1811/s1, Supplementary S1: Outpatient pulmonary rehabilitation program.
